# Lifestyle factors, glycemic traits, and lipoprotein traits and risk of liver cancer: a Mendelian randomization analysis

**DOI:** 10.1038/s41598-024-59211-3

**Published:** 2024-04-12

**Authors:** Honglu Zhang, Jiyong Liu

**Affiliations:** 1 Department of Pharmacy, Fudan University Shanghai Cancer Center, Shanghai, China; 2 Department of Oncology, Shanghai Medical College of Fudan University, Shanghai, China

**Keywords:** Mendelian randomization, Liver cancer, Glycemic traits, Lipoprotein traits, Cancer epidemiology, Cancer screening, Risk factors, Genome

## Abstract

The current state of knowledge on the relationship between lifestyle factors, glycemic traits, lipoprotein traits with liver cancer risk is still uncertain despite some attempts made by observational studies. This study aims to investigate the causal genetic relationship between factors highly associated with liver cancer incidence by using Mendelian randomization (MR) analysis. Employing MR analysis, this study utilized previously published GWAS datasets to investigate whether lifestyle factors, glycemic traits, and lipoprotein traits would affect the risk of liver cancer. The study utilized three MR methods, including inverse variance-weighted model (IVW), MR Egger, and weighted median. Furthermore, MR-Egger analyses were performed to detect heterogeneity in the MR results. The study also conducted a leave-one-out analysis to assess the potential influence of individual SNPs on the MR analysis results. MR-PRESSO was used to identify and remove SNP outliers associated with liver cancer. MR analyses revealed that 2-h glucose (odds ratio, OR 2.33, 95% confidence interval, CI 1.28–4.21), type 2 diabetes mellitus (T2DM, OR 1.67, 95% CI 1.18–2.37), body mass index (BMI, OR 1.67, 95% CI 1.18–2.37), waist circumference (OR 1.78, 95% CI 1.18–2.37) were associated with increased risk of liver cancer. On the contrary, apolipoproteins B (APOB, OR 0.67, 95% CI 0.47–0.97), and low-density lipoprotein (LDL, OR 0.62, 95% CI 0.42–0.92) were negatively related to liver cancer risk. Additionally, after adjusting for BMI, apolipoproteins A-I (APOA-I, OR 0.56, 95% CI, 0.38–0.81), total cholesterol (TC, OR 0.72, 95% CI, 0.54–0.94), and total triglycerides (TG, OR 0.57, 95% CI, 0.40–0.78) exhibited a significant inverse correlation with the risk of liver cancer. This study supports a causal relationship between 2-h glucose, T2DM, BMI, and waist circumference with the increased risk of liver cancer. Conversely, the study reveals a cause-effect relationship between TC, TG, LDL, APOA-I, and APOB with a decreased risk of liver cancer.

## Introduction

Liver cancer ranks fourth among the leading causes of cancer-related mortality worldwide. The incidence of liver cancer continues to rise annually, making it a major public health concern^1^. Hepatocellular carcinoma and cholangiocarcinoma are the most common types of liver cancer, comprising 75–85% and 10–15% of all cases, respectively^2^. As the principal metabolic organ of the human body, liver possesses the capacity to regulate nutrient intake, eliminate toxins, modulate metabolism, and maintain metabolic homeostasis. Tumorigenesis often coincides with alterations in metabolic pathways, leading to metabolic dysregulation^3^. Although an increasing number of treatments have been approved for liver cancer in the past decade, most of these treatments can only offer limited survival benefits due to the difficulty in diagnosing liver cancer at an early stage^4,5^. Therefore, the identification of new risk factors and biomarkers can help us better understand the underlying mechanisms of liver cancer, develop more effective and personalized treatments, and ultimately improve the prognosis of patients.

Traditional observational studies have identified several possible risk factors for liver cancer, such as obesity^6–8^, T2DM^9,10^, smoking^11^, and alcohol consumption^12,13^, while HDL, and coffee consumption were reported to be protective factors^13–15^. However, the results of traditional observational studies may be influenced by confounding factors. A meta-analysis of 24 cohorts indicates that T2DM is associated with an increased risk of liver cancer, but the results are significantly confounded by BMI and smoking^16^. Furthermore, most studies are unable to eliminate the potential interactions among risk factors, such as the correlation between BMI and blood lipid levels.

MR analysis, utilizing genetic variants robustly associated with exposures as instrumental variables, has emerged as a powerful approach to overcoming the confounding biases inherent in observational studies^17^. In our study, we employed MR analysis to investigate potential causal associations between the risk of liver cancer and several modifiable risk factors, including abdominal obesity (measured by waist circumference), overall obesity (measured by BMI), T2DM, lifestyle factors (such as smoking, alcohol consumption, and coffee intake), glycemic traits (such as 2-h glucose, FI, FG, and HbA1c), and lipoprotein traits (such as HDL, LDL, TC, TG, APOA-I, and APOB).

## Methods

### Study design

In this study, we conducted a MR analysis to explore the potential causal effects of obesity, T2DM, glycemic traits, and lipid traits on the risk of liver cancer. The genetic instruments for the exposures were retrieved from published genome-wide association studies (GWAS). The instrumental-variable MR analysis was employed to simulate randomized controlled trials in offspring by randomly allocating single nucleotide polymorphisms (SNPs). This approach allowed us to eliminate the potential confounding factors in our analysis and increase the validity of our findings.

### Exposure data

GWAS data for glycemic traits were obtained from the Meta-Analyses of Glucose and Insulin-related traits Consortium (MAGIC)^18^. The GWAS data included separate analyses for FG, FI, 2-h glucose concentration, and HbA1c among 200,622, 151,013, 112,283, and 146,806 participants, respectively. Adjustments for BMI, principal components, and study-specific covariates were made for each glycemic trait^18^. Similarly, the GWAS data for type 2 diabetes (T2DM) included 228,499 T2DM cases and 1,178,783 controls, and adjustments were made for age, gender, BMI, and the first ten genomic principal components^19^. Additionally, candidate genetic instruments for lipid traits were extracted from summary-level GWAS, which included data for HDL, LDL, TC, TG, APOA-I, and APOB^20^. Lastly, genetic variants associated with lifestyles, including smoking^21^, alcohol drinking^21^, coffee consumption^22^, waist circumference^23^, and BMI^23^, were obtained from corresponding GWAS. To reduce heterogeneity in the MR analysis, all GWAS data were sourced from population Caucasian. The methods used to measure the exposure data are shown in Supplementary Table S1.

### Outcome data

The study obtained summary-level GWAS data for liver cancer (malignant neoplasm of liver and intrahepatic bile ducts, defined by the International Classification of Diseases-Tenth Revision code C22) from the FinnGen consortium^24^. All FinnGen participants, including patients and control subjects, have provided informed consent for biobank research under the Finnish Biobank Act. Individuals with ambiguous gender, high genotype missingness (> 5%), excess heterozygosity (± 4 SDs), other cancers, and non-Finnish ancestry were excluded from this dataset.

### Genetic instrument selection

Genetic instruments for all exposures were selected at genome-wide significance threshold (p < 5 × 10^–8^) from corresponding GWAS. Linkage disequilibrium (LD) for each risk factor's SNPs was calculated using the PLINK clumping method with the 1000 Genomes LD reference panel for the European population. SNPs with an LD value of r^2^ greater than 0.01 and a clump window of less than 10 kb were excluded, with only the SNP with the lowest p-value being retained.

The relevant information was extracted: chromosome, effect allele (EA), other allele (OA), effect allele frequency (EAF), effect sizes (β), standard error (SE), and p-value. Subsequently, we calculated the explained variance (R^2^) and F-statistic parameters to determine whether the identified IVs were strongly associated with exposure. Generally, SNPs with F-statistic parameters > 10 are considered strong instruments^25^. In our study, R^2^ = 2 × EAF × (1 − EAF) × β^2^/(2 × EAF × (1 − EAF) × β^2^ + 2 × EAF × (1 − EAF) × N × SE^2^), where N is the sample size of the GWAS, and F = R^2^ × (N − 2)/(1 − R^2^)^26^.

### Mendelian randomization analyses

Three distinct methods of MR analysis were conducted in this study, including random-effects inverse-variance weighted (IVW), MR Egger, and weighted median. These methods were implemented to account for variant heterogeneity and the pleiotropy effect. IVW was considered the primary outcome due to its ability to combine the Wald ratio of each SNP on the outcome, the slope of the weighted regression of the SNP-outcome effects on the SNP-exposure effects (with an intercept of zero), and provide an estimated result. Weighted median allows the use of invalid instruments under the assumption that at least half of the instruments used in the MR analysis were valid^27^. To strengthen the IVW estimates, we employed both the weighted median and MR-Egger, as they offer more robust estimations a broader set of scenarios, despite producing broader CIs^28^. We also utilized MR-PRESSO to identify and remove SNP outliers associated with liver cancer.

For significant estimates, the horizontal pleiotropy was assessed by MR-Egger intercept test, and the Cochran's Q test was used to identify heterogeneity. Funnel plots are similar to the methods used to assess publication bias in meta-analysis and were used to assess possible directional pleiotropy. The causal relationship between exposure and outcome was investigated using leave-one-out analysis to determine if a single SNP influenced the relationship. The Multivariable IVW considers multiple exposure factors simultaneously. It limits the effects of SNP-exposure on their corresponding effects on the characteristics of other assumed risk factors along an indirect pathway by regaining the summary genetic associations resulting from genetic associations with exposure and risk factors in a weighted regression model^29^. A multivariable random-effects IVW model was used to adjust for BMI in the analysis of waist circumference, HDL, LDL, APOA-I, APOB, TC, and TG. All analyses were performed using the R package TwoSampleMR and MVMR in R version 4.3.0^30^.

Generally, IVW is more robust in statistical power than other MR methods^31^. The CI were calculated from the same equations that generated p-values. MR-Egger often had wider CI and non-significant p-values than IVW due to a loss of power, which was also evident in the present study. Consequently, IVW was the preferred method to screen for significant causal results^32^. However, the IVW estimates may be biased if horizontal pleiotropy existed. In this situation, it is advisable to consult the MR-Egger estimates, as this method adjusts the IVW analysis by accommodating the horizontal pleiotropic effect across all SNPs o be unbalanced or directional^33^. In addition, studies have emphasized the necessity of consistent beta direction across all MR approaches in most MR studies, which was also adhered to in our study^34,35^.

Utilizing a large number of cases from GWAS data and MR design, our study can simulate randomized controlled trials within an observational setting. Randomized controlled trials are recognized widely as a valid method for studying causality. However, they can be costly and often unfeasible to conduct. The MR approach mitigates confounding bias since SNPs are randomly assigned during conception, thus effectively circumventing the issue of reverse causality compared to observational studies.

## Results

**Figure 1:**
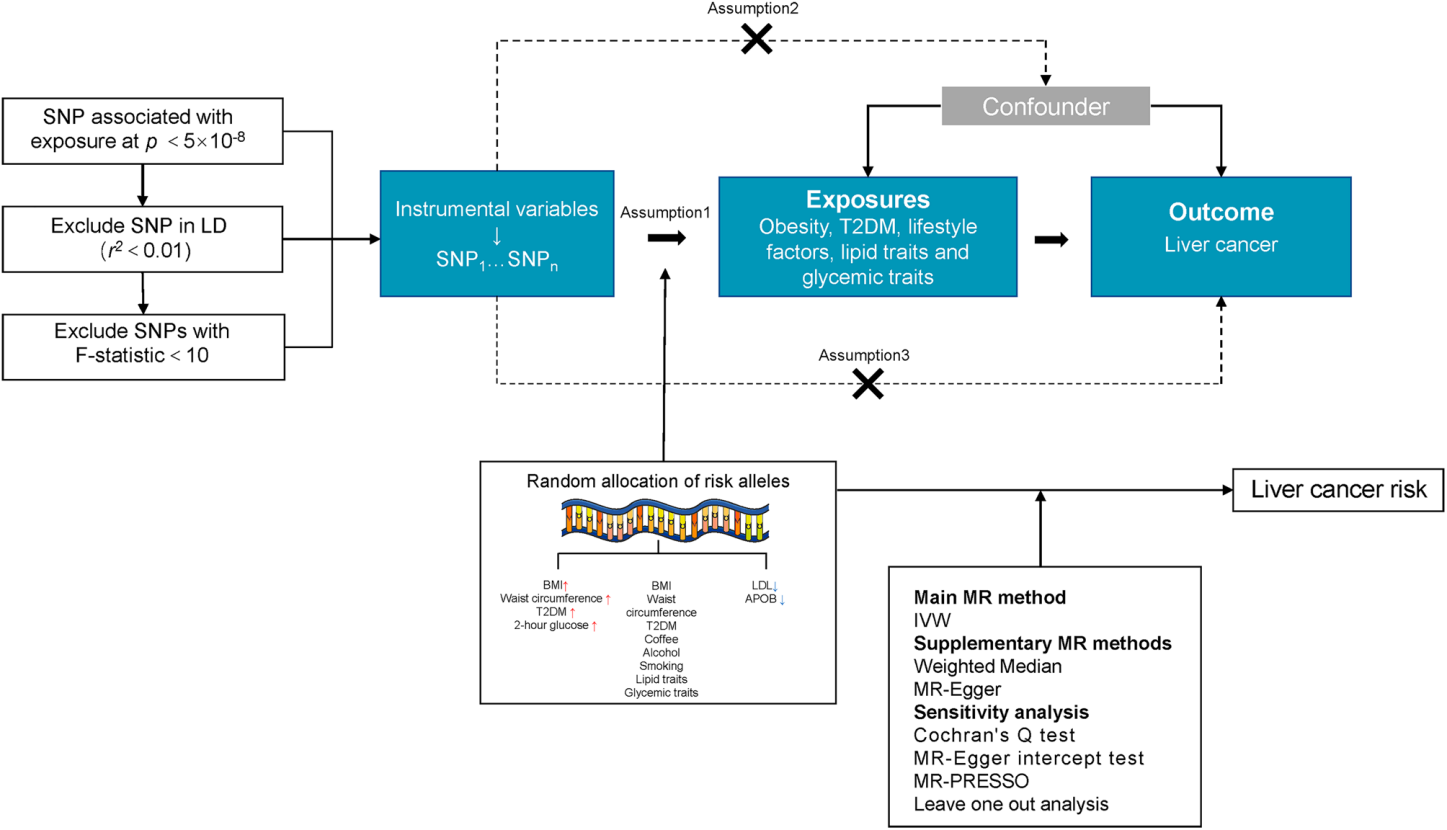
The study design and assumptions of the MR approach. Assumption 1 requires that the selected genetic variants, proposed as instrumental variables, are strongly associated with the risk factor under investigation. Assumption 2 states that these genetic variants should not be associated with potential confounding factors, while Assumption 3 asserts that these genetic variants should impact the outcome exclusively through the risk factor, rather than through alternative pathways. By reducing residual confounding and reverse causality, the MR approach can enhance the causal inference of the exposure-outcome association. The instrumental variables selected for studying the effects of modifying the exposure are randomly allocated at conception, thereby minimizing the susceptibility to confounding by environmental factors and reverse causation. These instrumental variables are used in the inverse-variance weighted (IVW) analysis to provide an estimate of the causal effect. Type 2 diabetes mellitus (T2DM) is a particular example of the exposure-outcome association being investigated.

**Figure 2:**
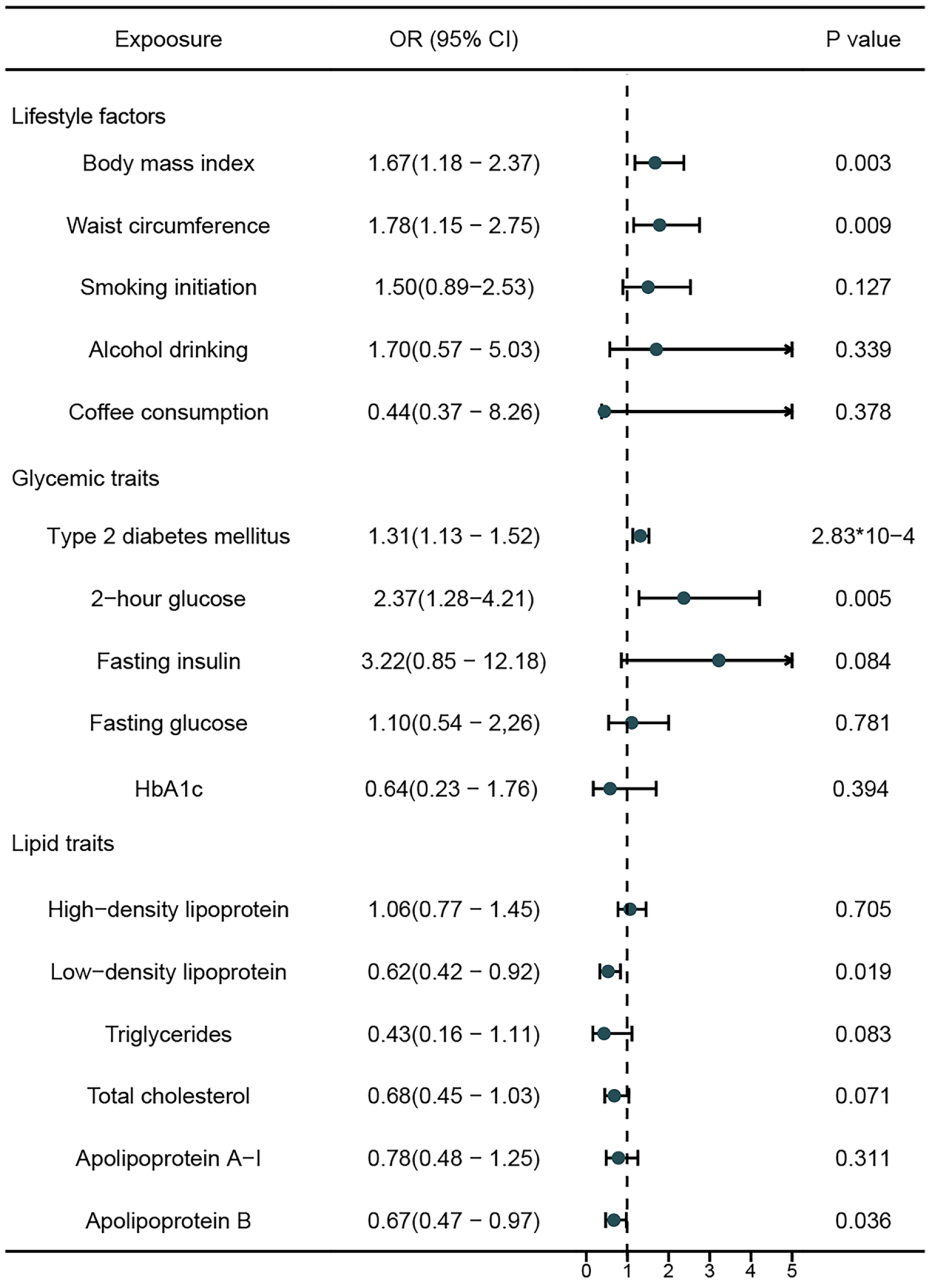
MR analysis results of the exposures to liver cancer risk.

An overview of the study design and the assumptions underlying MR analysis are illustrated in Fig. 1. The main information of SNPs, including effect allele, other allele, p value, beta, and standard error, was collected systematically for MR analysis. All genetic instruments in this study had F statistics greater than the conventional threshold of 10, signifying no evidence of weak instrument bias^36^. Table 1 contains detailed information on the GWAS data sources utilized in our study. Three MR analytical methods, IVW, weighted median, and MR Egger, were employed to estimate the causal effects of exposures on the risk of liver cancer. In IVW method, the ORs of liver cancer were 2.33 (95% CI 1.28–4.21) per 1-SD increase in 2-h glucose, 1.31 (95% CI 1.13–1.52) for 1-unit increase in log OR of T2DM, 1.67 (95% CI 1.18–2.37) per 1-SD increase in BMI, and 1.78 (95% CI 1.15–2.75) per 1-SD increase in waist circumference. Conversely, APOB (per 1-SD increase, OR 0.67; 95% CI 0.47–0.97) and LDL (per 1-SD increase, OR 0.62; 95% CI 0.42–0.92) decreased the risk for liver cancer (Fig. 2). The weighted median and MR egger results showed a consistent direction with IVW analysis. In contrast to the results of many observational studies, we did not find causal relationship between smoking initiation, alcohol drinking, coffee consumption with the risk of liver cancer.

**Table 1:** Detailed Information on Used GWAS data source.

Exposure	Unit	Adjustments	IV-SNPs	Sample size	Variance explained		F-statistic	PubMed ID or URL
Body mass index	SD	Age and any necessary study-specific covariates	458	461,460		5.7%	61.26	UK Biobank
Waist circumference	SD	Age and study-specific covariates	374	462,166		3.4%	44.15	UK Biobank
Smoking initiation	SD in prevalence of smoking initiation	Age, sex, and the first 10 genetic principal components	93	607,291		2.5%	166.06	30643251
Alcohol drinking	SD increase of logtransformed alcoholic drinks/per week	Age, sex, and the first 10 genetic principal components	76	941,280		0.5%	69.74	30643251
Coffee consumption	50% change	Age, sex, BMI, total energy, proportion of typical food intake, and 20 genetic principal components	29	375,833		0.3%	38.99	31046077
Type 2 diabetes mellitus	1-unit in log odds ratio of type 2 diabetes	Age, sex, and the first 10 geneti principal components	279	1,407,282		25.8%	1758.4	32541925
2-h glucose	SD	BMI, study-specific covariates, and principal components	14	112,283		2.6%	214.41	34059833
Fasting insulin	SD	BMI, study-specific covariates, and principal components	38	151,013		0.4%	11.86	34059833
Fasting glucose	SD	BMI, study-specific covariates, and principal components	70	200,622		1.2%	20.38	34059833
HbA1c	SD	BMI, study-specific covariates, and principal components	75	146,806		0.7%	13.79	34059833
High-density lipoprotein	SD	Lipid-lowering medication, and principal components	362	403,943		10.1%	124.74	32203549
Low-density lipoprotein	SD	Lipid-lowering medication, and study-specific covariates	82	201,678		6.8%	179.44	UK Biobank
Triglycerides	SD	Lipid-lowering medication, and study-specific covariates	12	21,545		5.1%	95.24	27005778
Total cholesterol	SD	Lipid-lowering medication, and study-specific covariates	23	21,491		9.4%	101.47	27005778
Apolipoprotein A-I	SD	Lipid-lowering medication, and study-specific covariates	11	20,687		4.7%	93.31	27005778
Apolipoprotein B	SD	Lipid-lowering medication, and study-specific covariates	21	20,690		8.9%	98.24	27005778
Malignant neoplasm of liver and intrahepatic bile ducts	–	Age, sex, 10 genetic principal components, and genotyping batch	–	309,174		–	–	FinnGen consortium

**Figure 3:**
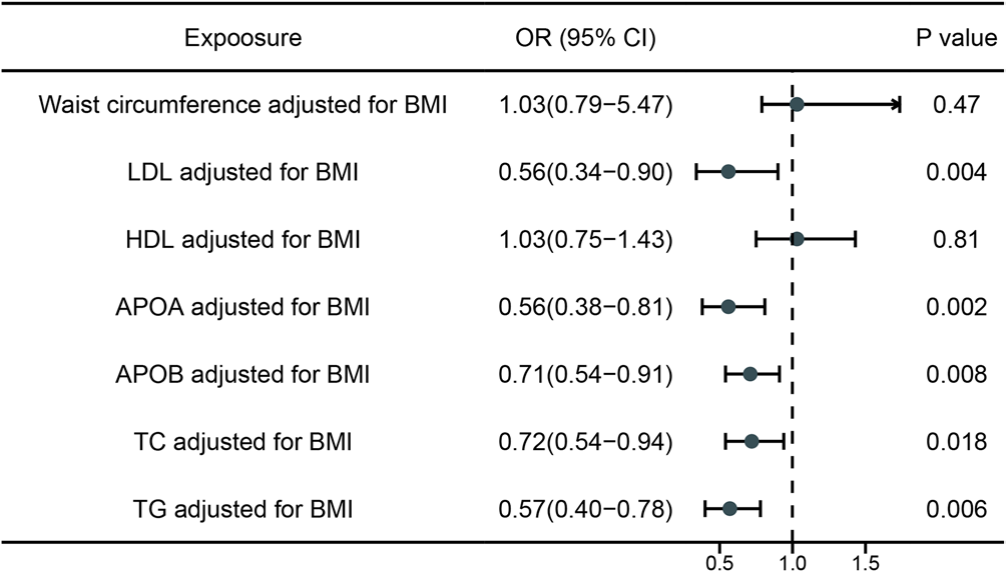
Genetically predicted BMI-adjusted associations of waist circumference and lipid traits.

Subsequently, we carried out a multivariable Mendelian randomization (MVMR) analysis of waist circumference and blood lipid traits with adjustment for BMI. Following the adjustment, the inverse association between APOB and LDL levels and the risk of liver cancer became stronger, while waist circumference was found to be unrelated to liver cancer risk. Furthermore, after adjusting for BMI, APOA (0.56, 95% CI 0.38–0.81), TC (0.72, 95% CI 0.54–0.94), and TG (0.57, 95% CI 0.40–0.78) displayed a significant inverse correlation with the risk of liver cancer (Fig. 3).

Moreover, to ensure the validity of the above findings, a range of sensitivity analyses were conducted. These included the Cochran's Q test, MR Egger intercept test, and MR-PRESSO global test. (Table 2). All* P* values of the MR-Egger intercept tests were > 0.05, indicating that no horizontal pleiotropy existed. Cochran's Q test analysis revealed heterogeneity in TC, TG, HDL, and LDL. Nevertheless, as random effects IVW can balance pooled heterogeneity, the recorded heterogeneity was deemed acceptable^25^. The Egger intercepts did not detect any pleiotropy, indicating that pleiotropic bias was not introduced to MR estimates in the presence of heterogeneity. Furthermore, after removing outliers in the MR-PRESSO analysis, the associations remained constant, and no variation was observed in the estimates before and after outlier elimination. The scatter plots, funnel plots and the results of leave-one-out analysis were showed in Supplementary Fig. 1–3.

**Table 2:** Sensitivity analysis of the MR analysis results of exposures and outcomes.

Exposure	Cochrane’s Q		MR-Egger		MR-PRESSO
	Q value	P	Intercept	P	P
Body mass index	409.72	0.49	− 0.003	0.75	0.62
Waist circumference	339.56	0.54	− 0.013	0.16	0.59
Smoking initiation	84.34	0.43	− 0.008	0.80	0.55
Alcohol drinking	53.30	0.95	− 0.047	0.06	0.94
Coffee consumption	26.41	0.49	0.017	0.49	0.54
Type 2 diabetes mellitus	578.51	0.02	− 0.007	0.24	0.004
2-h glucose	5.34	0.72	− 0.055	0.37	0.68
Fasting insulin	27.06	0.85	− 0.016	0.63	0.85
Fasting glucose	61.27	0.57	0.017	0.26	0.52
HbA1c	60.57	0.78	0.022	0.14	0.79
High-density lipoprotein	405.61	< 0.001	− 0.010	0.15	0.001
Low-density lipoprotein	120.56	< 0.001	0.019	0.18	0.001
Triglycerides	80.24	< 0.001	0.164	0.36	0.001
Total cholesterol	60.18	< 0.001	8.21 × 10^–5^	0.99	0.24
Apolipoprotein A-I	12.43	0.13	0.066	0.48	0.21
Apolipoprotein B	38.21	< 0.001	0.014	0.76	0.007

## Discussion

This study implemented several MR approaches to appraise the possible causal association of BMI, waist circumference, lifestyle factors, glycemic traits, and lipoprotein traits with liver cancer. We demonstrated that abdominal obesity (measured by waist circumference), overall obesity (measured by BMI), T2DM, and 2-h glucose causally increased the risk of liver cancer. A possible causal association is observed between APOB and LDL with decreased risk of liver cancer. After adjusting for the impact of BMI, we identified a reverse causal relationship between TC, TG, APOA-I, with the liver cancer risk. There is no evidence that FG, FI, HbA1c, HDL, smoking initiation, alcohol drinking, and coffee consumption are causally associated with liver cancer.

Many experimental and observational epidemiological studies have investigated that obesity was associated with an ascended risk of liver cancer^7,37,38^. In a meta-analysis of 11 cohort studies with a total of 11,079 cases, overweight and obesity were associated with a 17% and 89% higher risk of liver cancer respectively, which is consistent with our findings^8^. In a systematic review and meta-analysis, waist circumference is a significant independent risk factor related to the incidence of liver cancer^39^. By two-sample MR analysis, we have confirmed the causal relationship between BMI, waist circumference with liver cancer risk. However, after adjusting for BMI, waist circumference lost its causal relationship with the risk of liver cancer. This suggests that abdominal obesity may not be a significantly higher risk factor compared to overall obesity. Several potential mechanisms may explain the association between obesity and liver cancer, including a high level of proinflammatory cytokines^40^, disorders of adipose tissue metabolism^41^, and abnormal levels of hormones^42^.

Prospective cohort studies have reported a significant association between T2DM and a higher risk of developing liver cancer^9,43^. According to a population-based case–control study, diabetic individuals were found to have a 2.87-fold higher risk of liver cancer compared to non-diabetic controls, independent of viral hepatitis, alcoholic liver disease, or nonspecific cirrhosis^44^. Our MR study strengthened the causal nature of this positive association. Moreover, an umbrella review of meta-analyses of prospective studies examined prediabetes symptoms, such as impaired FG and glucose tolerance, which were associated with the incidence of liver cancer^45^.

In addition, a cohort study that examined 1,140,000 Australians found an increased risk of liver cancer in association with glucose tolerance^46^. Consistent with the previous studies mentioned, our investigation demonstrated robust MR associations between liver cancer risk and glucose tolerance (measured by 2-h glucose) in two independent datasets. This indicates that impaired glucose tolerance may serve as a risk factor for liver cancer and is beneficial for predicting the risk of liver cancer before patients are diagnosed with diabetes. Impaired glucose tolerance and T2DM have various factors that may contribute to the initiation and progression of liver cancer, including insulin/insulin-like growth factor-related factors^47^, proinflammatory cytokines^48^, gut microbiota dysbiosis^49^, and angiogenesis^50^. However, our study did not find causal associations of HbA1c, FG, and FI with liver cancer risk, which differs from observational studies^51,52^.

So far, the relationship between lipoprotein characteristics and liver cancer remains unclear. In most cases, the available studies have insufficient cases of liver cancer to explore possible associations with lipoprotein traits independently^53,54^. A recent MR study showed that lower levels of LDL were linked with an increased risk of total cancer^55^. In our MR study, we found a reverse causal relationship between LDL and the risk of liver cancer. As the apolipoprotein of LDL, the decrease of APOB level has also been identified as a causal risk factor for liver cancer. After adjusting for BMI by MVMR, the reverse relationship between LDL, APOB with the risk of liver cancer was further strengthened. Therefore, our findings may indicate that contrary to people's expectations, low LDL level may not confer benefits and could potentially be detrimental. Some underlying mechanisms in support of APOB, LDL and the risk of liver cancer have been proposed. A study of comparative systems genomics demonstrated low-APOB activity was associated with upregulation of oncogenic and metastatic regulators (CD44, FOXM1, ERBB2), inhibition of tumor suppressors, (PTEN and TP53), and also associated with poor prognosis^56,57^.

As blood lipid characteristics are often closely related to obesity, analyzing these characteristics independently of BMI has important clinical value in assessing the risk of liver cancer. After adjusting for BMI through MVMR, our study found a negative causal relationship between TC and TG with the risk of liver cancer. Similarly, a large retrospective study over multiple years has shown that low TC and TG concentration are independent risk factors for HCC^58^. Although we currently do not understand the exact mechanism, several potential mechanisms can explain the association. Firstly, cholesterol imbalance itself may be a part of liver cancer occurrence. Various inflammatory factors that affect the occurrence and progression of cancer, including IL-6, IL-1, and TNF-a dysregulation, can all affect cholesterol synthesis^59^. In addition, an animal study found that high cholesterol levels can enhance the tumor-killing effect of NK cells. This finding demonstrates a new role for cholesterol in affecting immune factors^60^.

This suggests that for obese populations, weight loss may be a more effective way to reduce the risk of liver cancer than regulating blood lipid levels. Additionally, after adjusting for BMI, we found that APOAI is a protective factor for liver cancer, while there is no causal relationship between HDL and the risk of liver cancer. Further research may be needed to explain the underlying mechanisms.

Epidemiological data on the association between light alcohol drinking and liver cancer are inconsistent^61–63^. However, heavy alcohol drinking (> 50 g per day) showed a dose–response relationship with liver cancer^12,63^. Our study did not confirm the dose–response association but could not exclude a possible weak nonlinear association of alcohol drinking with liver cancer. Likewise, no causal association between coffee consumption and liver cancer risk was found in our study. In a meta-analysis of 18 cohorts, an extra two cups of coffee were associated with a decreased risk of hepatocellular carcinoma (OR 0.73, 95% CI 0.63–0.85)^64^. Given the limited data and potential publication bias examining the link from coffee consumption to liver cancer, more study is needed.

Several limitations should be considered in the present study. Our participants were solely of European ancestry, therefore, extrapolation of our findings to other ethnicities must be approached with caution, given possible cultural and lifestyle differences. Additionally, the MR approach is limited by potential pleiotropy, including vertical and horizontal pleiotropy^65,66^.

However, the pleiotropic should not impact the validity of our findings for two reasons. Firstly, our MR-Egger intercept test revealed limited evidence of vertical pleiotropy in most of the associations analyzed in our MR study. Secondly, for associations showing significant evidence of horizontal pleiotropy, MR-PRESSO analysis identified only a small number of outliers, and the results remained consistent or even became more robust after removing these outliers. However, pleiotropy should not compromise the validity of our findings for two reasons. First, our MR-Egger intercept test showed low evidence of vertical pleiotropy in most of the associations examined in our MR study. Second, the MR-PRESSO analysis identified a few outliers for associations that showed significant evidence of horizontal pleiotropy. These outliers were removed, resulting in consistent or even more robust outcomes.

Moreover, our estimations could be prone to selection bias and impacts of unmeasured environmental factors. For instance, individuals identified with a high polygenic risk score concerning circulating lipids are at a greater risk of leaving the cohort due to their increased vulnerability to chronic cardiovascular diseases. Additionally, we couldn't regulate the impact of medication used for glucose-lowering and lipid-lowering purposes.

## Conclusion

In summary, this MR study suggests causal roles of abdominal obesity, overall obesity, T2DM, and two-hour glucose in the development of liver cancer. On the contrary, genetically determined higher TC, TG, LDL, APOA-I, APOB level is associated with a lower risk of liver cancer. Further studies are required to explore the underlying mechanisms of the causal relationships explored by our study.

### Supplementary Information

Supplementary Information.

## Data Availability

All the GWAS data used for this study are publicly available and their original studies are shown in Table 1.

## Abbreviations

APOA-I: Apolipoproteins A-I
APOB: Apolipoproteins B
BMI: Body mass index
CI: Confidence interval
FG: Fasting glucose
FI: Fasting insulin
HDL: High-density lipoprotein
IVW: Inverse variance-weighted model
LDL: Low-density lipoprotein
MR: Mendelian randomization
OR: Odds ratio
TC: Total cholesterol
TG: Total triglycerides
T2DM: Type 2 diabetes mellitus
