# Supplementary material for: Lifestyle factors, glycemic traits, and lipoprotein traits and risk of liver cancer: a Mendelian randomization analysis
Source: Sci Rep. 2024 Apr 12;14:8502. doi: 10.1038/s41598-024-59211-3 (PMC11009263; doi:10.1038/s41598-024-59211-3)
Supplement: Supplementary file 1 — Supplementary Information. [file 41598_2024_59211_MOESM1_ESM.docx]

**Supplementary Table S1** The methods used to measure the exposure data

| Exposure | Description |
| --- | --- |
| Glycemic Traits | Analyses included data for FG and 2hGlu measured in mmol/l, FI measured in pmol/l, and HbA1c in %. Reported use of diabetes-relevant medication(s) or had a FG ≥7 mmol/L, 2hGlu ≥11.1mmol/L, or HbA1c ≥ 6.5%. 2hGlu measures were obtained 120 minutes after a glucose challenge in an oral glucose tolerance test (OGTT). Measures for FG and FI taken from whole blood were corrected to plasma level using the correction factor 1.13. |
| Lipoprotein Traits | All traits were analyzed using the same high-throughput serum NMR metabolomics platform in the same analysis laboratory. HDL, LDL, TC, and TG were measured in mmol/L. APOA-I, APOB were measured in g/L. |
| BMI | Body mass index (BMI: weight [in kilograms] / height [in meters]^2^) was corrected for age and genomic principal components as well as any additional study-specific covariates (e.g. recruiting center), in a linear regression model. For studies with non-related individuals, residuals were calculated separately by sex, whereas for family-based studies sex was included as a covariate in the model. Additionally, residuals for case/control studies were calculated separately. Finally, residuals were subject to inverse normal transformation. |
| Waist circumference | The ratio of waist circumference adjusted for age and study-specific covariates if necessary. For each cohort, residuals were calculated for men and women separately and then transformed by the inverse standard normal function. |
| Alcohol drinking | 1. Defined as the average number of drinks a participant reported drinking each week, aggregated across all types of alcohol. If a study recorded binned response ranges (e.g., 1-4 drinks per week, 5-10 drinks per week) we used the midpoint of the range. For example, if an individual reported 1-5 drinks per week, we assume they drank 2.5 drinks per week on average.  2. This was measured in a variety of ways.  a. In the past week, how many alcoholic beverages did you have?  b. Thinking about the past year, on the average how many drinks did you have each week?  3. This phenotype was left-anchored at 1 and log-transformed prior to analysis, in order to prevent outliers from having undue leverage on analyses. |
| Smoking initiation | 1. This is a binary phenotype. Any participant reporting ever being a regular smoker in their life (current or former) were coded “2”, while any participant who reported never being a regular smoker in their life were coded “1”.   2. Does not include information about pipes/cigar/chew, or other non-cigarette forms of tobacco use.  3. This phenotype was measured in a variety of ways.  a. Have you smoked over 100 cigarettes over the course of your life?  b. Have you ever smoked every day for at least a month?  c. Have you ever smoked regularly? |
| Coffee consumption | ACE touchscreen question "How many cups of coffee do you drink each DAY?"  The following checks were performed:  If answer < 0 then rejected  If answer > 99 then rejected  If answer > 10 then participant asked to confirm  If the participant activated the Help button they were shown the message:  Please provide an average considering your intake over the last year.If you are unsure, please provide an estimate or select Do not know. |
| Type 2 diabetes mellitus | ICD-9-CM diagnosis codes from electronic health care records were available for The Million Veteran Program(MVP) participants from as early as 1998. Participants were classified as a T2D case if they had 2 or more T2D-related diagnosis codes (ICD-9-CM 250.2x) from VA or fee basis inpatient stays or face-to-face primary care outpatient visits in the 731 days before the enrollment date up to July 1st of 2017, excluding those with co-occurring diagnosis codes for T1D (250.1x), secondary or other diabetes or a medical condition that may cause diabetes (249.xx). Participants were selected as controls if they had no ICD-9-CM diagnosis code for type 1, type 2, or secondary diabetes mellitus up to July 2017. |


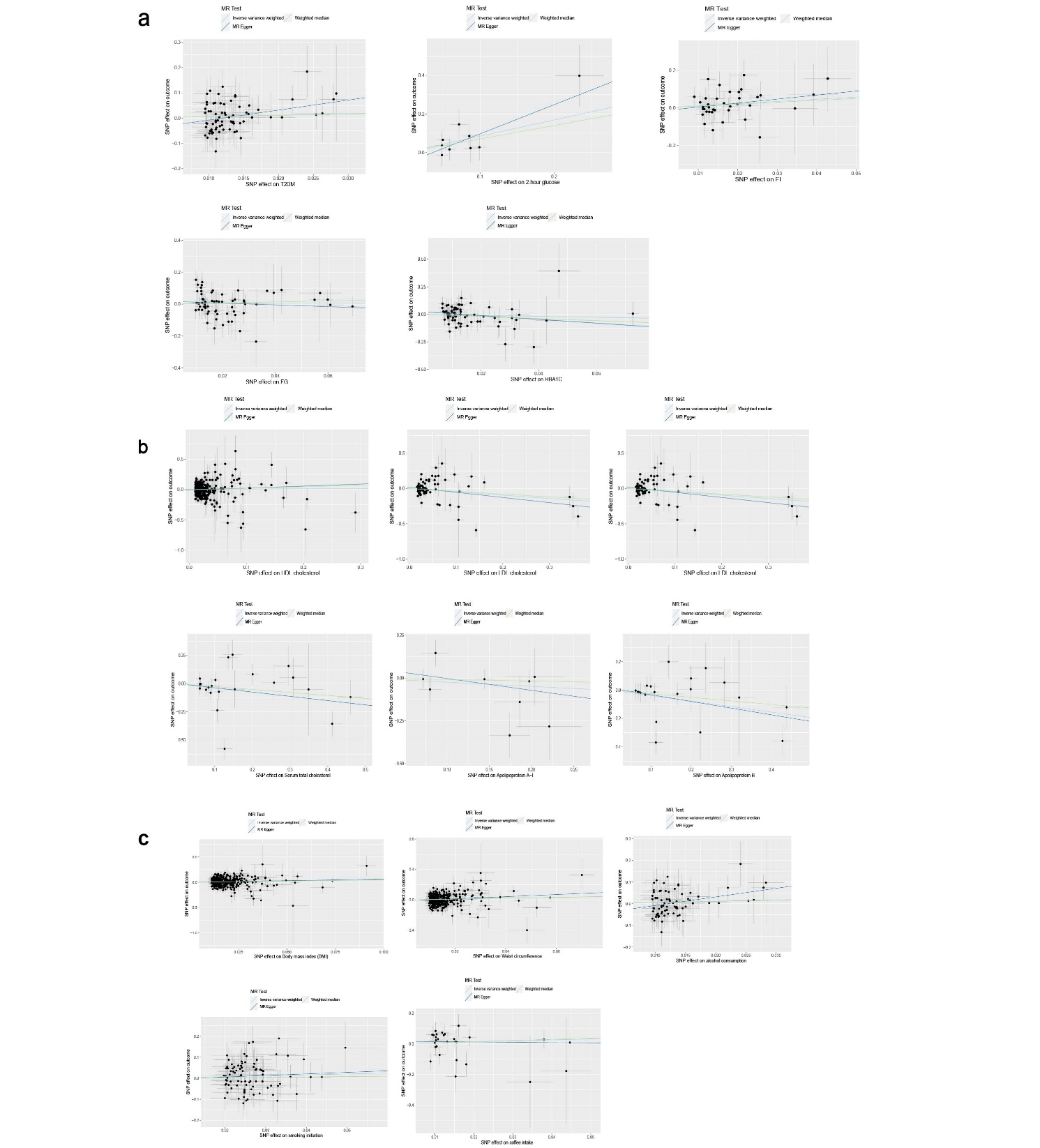


**Supplementary Figure S1** MR-Egger estimates of scatter plots from exposure to liver cancer. (a) glycaemic traits; (b) lipid traits; (c) lifestyle factors.


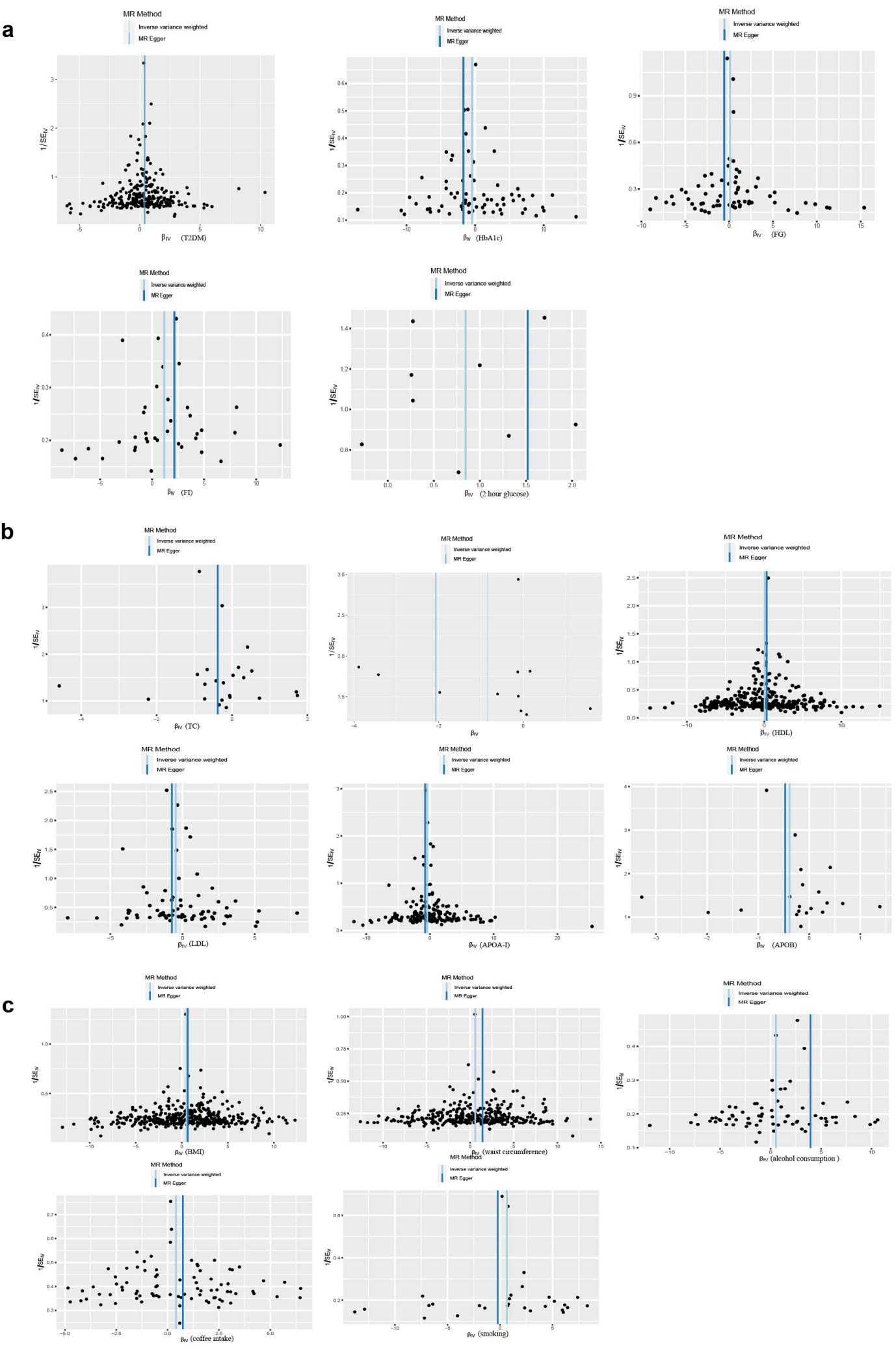


**Supplementary Figure S2** Funnel plots of MR analyses from exposures on liver cancer. (a) glycaemic traits; (b) lipid traits; (c) lifestyle factors.


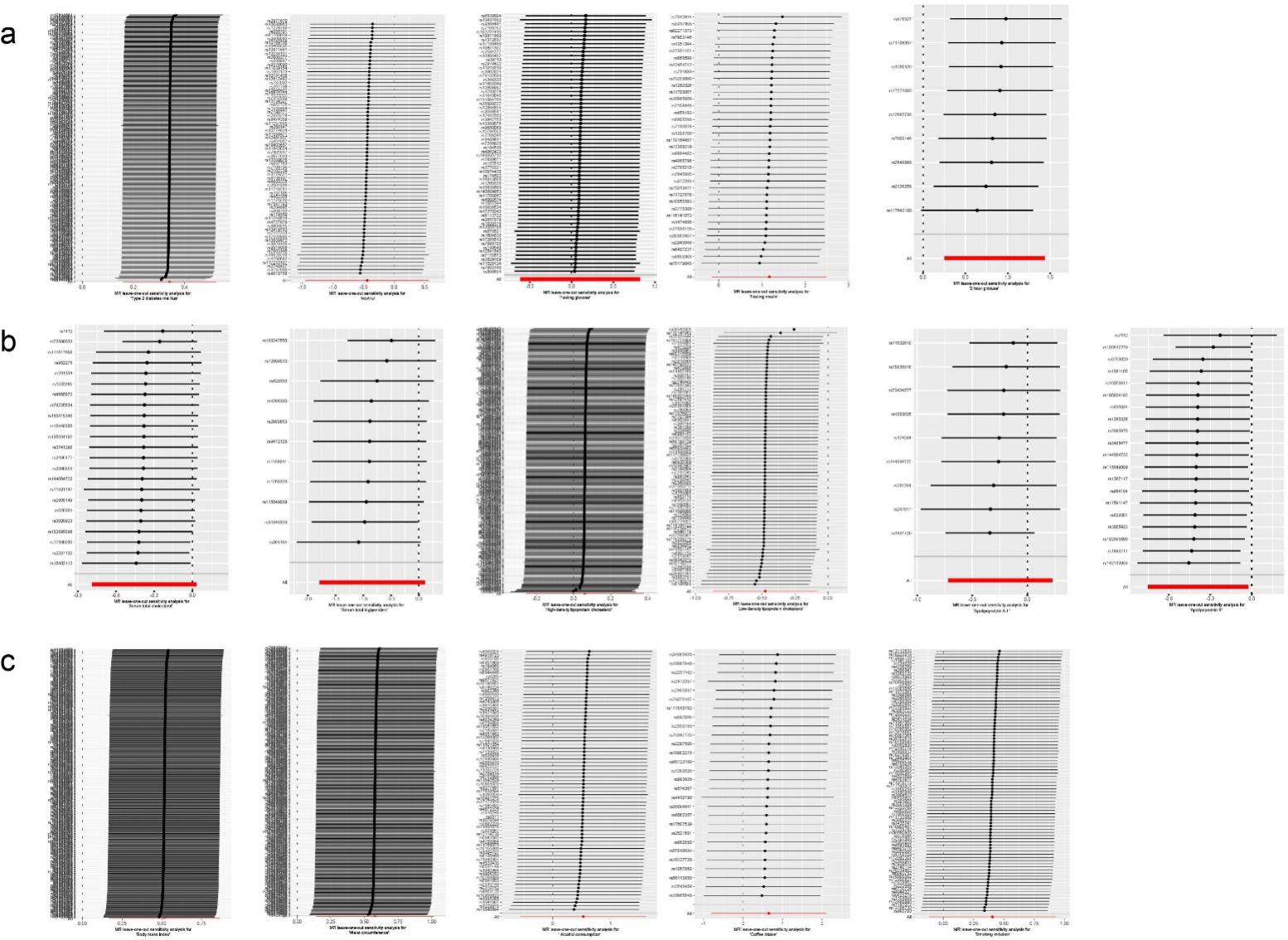


**Supplementary Figure 3** Leave one out analysis of MR analyses from exposures traits on liver cancer. (a) glycaemic traits; (b) lipid traits; (c) lifestyle factors.
